# A new measure for infant mental health screening: development and initial validation

**DOI:** 10.1186/s12887-016-0744-1

**Published:** 2016-12-01

**Authors:** Janni Ammitzbøll, Bjørn E. Holstein, Lisbeth Wilms, Anette Andersen, Anne Mette Skovgaard

**Affiliations:** 1 National Institute of Public Health, University of Southern Denmark, Øster Farimagsgade 5a, 2., 1353 Copenhagen K, Denmark; 2 The Municipality of Gentofte, 2920 Charlottenlund, Denmark; 3 Department of Public Health, University of Copenhagen, Øster Farimagsgade 5, P.O. Box, 1014, Copenhagen K, Denmark

**Keywords:** Mental health, Infancy, Screening instrument, Community, Health services

## Abstract

**Background:**

Mental health problems are a major public health challenges, and strategies of early prevention are needed. Effective prevention depends on feasible and validated measures of screening and intervention. Previous research has demonstrated potentials for infant mental health screening by community health nurses (CHN) in existing service settings in Denmark. This study was conducted to describe the development of a service setting based measure to screen for infant mental health problems, to investigate problems identified by the measure and assess the validity and feasibility in existing public health settings.

**Methods:**

Experts within the field developed a short, feasible and comprehensive measure. A consecutive sample of 2973 infants from 11 municipalities around the city of Copenhagen was screened at 9–10 months. Face validity and feasibility were evaluated among CHNs. Data on child and family factors and the results of screening were included in descriptive analyses. Exploratory factor analysis (EFA) was used to assess content validity.

**Results:**

The measure identified problems of communication and interaction in 20.7% of the children, problems of eating in 20.1%, attention problems in 15.9% and problems of emotional regulation in 14.3%. Significant gender differences were seen. EFA demonstrated that among 27 items 11 items were clustering into five areas: Problems of eating, emotions, attention, language and communication and attachment, respectively. High face validity and feasibility was demonstrated, and the participation was 91%.

**Conclusions:**

The new measure shows potentials for infant mental health screening. However, further exploration of construct validity and reliability is needed.

## Background

Mental health problems in childhood are a major challenges to public health [1], being the most frequent causes of learning disabilities and social impairment, and with a high risk of persistency into adolescence and adult age [2, 3]. Solid evidence points to onset before the age of three regarding neuro-developmental disorders, e.g. autism spectrum disorders (ASD) and attention deficit hyperactivity disorders (ADHD), whereas severe emotional and behavioral disorders e.g. disorders of attachment may have their onset in the first year of living [2, 3].

Preventive strategies including the general population are recommended as the most effective strategy to reduce the overall burden of mental illness in childhood [4, 5]. Prevention targeting the earliest symptoms of psychopathology has the highest probability of significantly reducing risk of progression of impairing symptoms and development of academic and socio-emotional complications [5, 6].

Measures to identify symptoms of psychopathology in early childhood comprise tools developed for clinical settings or as screening measures for children at risk. With regard to non-clinical populations, the screening measures published have mainly concerned developmental problems, early detection of autism and attention deficit disorders; or screening for children’s socio-emotional and behavioral problems and family psychosocial problems, e.g. parental mental health problems, parental stress or parent–child relationship problems [4, 6]. Also measures developed for the mental health screening of older children have been extended downward to the age of 18 and 36 months [4].

So far, no measures have been published, that cover the full range of developmental psychopathology in the youngest children, e.g. children below the age of 12 months [4] and which has been demonstrated to be feasible in the general child health surveillance [5–7].

A Danish general population birth cohort study investigated early predictors of childhood mental health problems and the possibilities of screening within the existing service settings provided by community health nurses, (CHN) [8]. Potentials of the CHNs’ identification of early symptoms of preschool autism spectrum disorders (ASD), attention deficit disorders (ADHD) and symptoms of eating disturbances and functional somatic symptoms were suggested [9–11]. However, the study concluded that standardized measures were needed to ensure the validity [8, 12].

The objective of the present study was to develop a measure, specifically designed to identify mental health problems in infants from the general population, i.e. being feasible within the existing service settings delivered by CHNs.

The aims of this paper are 1) to describe the development of the screening measure, 2) to investigate the patterns of infant mental health problems identified by this measure, 3) to assess the validity and feasibility in existing public health settings.

## Methods

### Setting

The study was conducted within the existing child health surveillance in 11 urban and suburban municipalities around the city of Copenhagen. In Denmark all registered delivered childbirths are reported from midwives to the CHNs in the municipalities; and all families with newborn babies are offered a series of home visits free of charge by the same CHN. The CHN is a registered nurse with specific training in assessment of child health and development and communication with parents. This service provision is well accepted by parents, and more than 90% of infant families participate. The CHNs’ services involves scheduled home visits at child ages 1–5 weeks, 2–3 weeks, 4–6 months, and 8–10 months during the first year of life, and an assessment at school entry; however highly flexible according to the need of the individual child. In the study area, a standardized CHN record has been in use since 2002 [12], whereby information obtained by CHNs at home visits is systematically recorded in a clinical database, the Child Health Database (CHD). The database comprises information on child health and development, parent–child relations and the family situation recorded from the birth of the child and onwards [13–15].

### Development of a new measure

The measure was founded on theoretical and empirical knowledge on developmental psychopathology in young children, and created to fulfill the requirements to population based screening [4], be easy-to-use and well accepted by the parents and CHNs. Fundamentally, the measure should demonstrate sufficient validity and reliability, and be feasible in combination with intervention towards problems identified.

Building on the international literature of early developmental psychopathology [6, 16] and findings from a recent study embedded in existing primary health care service settings in Denmark [8, 14], we considered child age 9–10 months to be a window of opportunity regarding the CHNs screening of developmental problems as well as socio-emotional problems.

The items of the measure were created after reviewing the literature on validated measures to assess infant mental health problems, e.g.: 1) developmental tests, such as The Bayley’s Scales of Infant Development [17], 2) measures of child development based on parents’ reports, such as the Ages and Stages Questionnaire (ASQ) [18], 3) measures to assess particular problems, e.g. regulatory problems, such as the Infant Toddler Symptom Checklist (ITSCL) [19], or socio-emotional problems, such as the Infant Toddler Socio-Emotional Assessment (ITSEA) or Brief Infant Toddler Socio-Emotional Assessment (BITSEA) [20].

An expert group of developmental psychologists and child psychiatrists specialized in infant and toddler psychiatry, and experienced CHNs developed the measure, while including the following: 1) the theoretical frame of infants social and emotional development and developmental psychopathology [21], 2) the conceptualization of infant and toddlers’ mental health problems and disorders in the International Classification of Diseases (ICD-10) and the Diagnostic Classification Zero-To-Three-Revised Version, DC:0-3R [22, 23], 3) results from previous research [8, 14], 4) psychometric tools developed into the field [4, 6], and 5) empirical evidence and experiences of professionals working with infants and toddlers in public health and clinical settings. Basically, it was conditioned that the instrument could be easily applied in the procedures at home visits and perceived by the CHNs as appropriate and beneficial [4].

Taking these aspects into consideration, the measure was designed to be comprehensive, and include all areas of infants’ mental health addressed by CHNs in existing routines, but also short and feasible in existing routines. Among the scheduled home visits, the visit at child age 9–10 months was considered to be optimal regarding valid identification of mental health problems [8, 12, 14, 16].

The new measure, now called the Copenhagen Infant Mental Health Screening, comprises a one-page questionnaire with a total of 27 items that cover infants feeding and eating, sleep, contact and communication, language development, and the child’s social-emotional functioning. A short descriptive text accompanied each item and a written detailed manual provided definitions and more detailed descriptions and guidelines for use (Table 1).

**Table 1:** The Copenhagen Infant Mental Health Screening (CIMHS)

Domain	Item	Description		
Sleep regulation	Stable sleeping pattern	The child has established a steady pattern for sleeping and being awake	Yes	no
	Falling asleep time	The child falls asleep within one hour	Yes	no
	Interrupted sleep	The child is able to sleep at least three consecutive hours	Yes	no
Eating	Appetite regulation	The child indicates clearly when it is hungry or full	Yes	no
	Eats too little	The child has to be pressured to eat enough	Yes	no
	Refusal to eat	The child refuses food even though it has not eaten for a long time	Yes	no
	Vomiting without otherwise being ill	The child vomits more than once a week	Yes	no
Expression of emotions	Generally happy and satisfied	The child is happy and satisfied more than 80% of its waking time	Yes	no
	Often irritable, fussy, dissatisfied	The child has at least two episodes every day where it is irritable, fussy, dissatisfied	Yes	no
	Cries often	The child cries more than one hour every day	Yes	no
	Emotionally blunted	The child shows no happiness, has limited facial expression and seems sad more than 50% of its waking time	Yes	no
Curiosity and interest	Curiosity, exploring	The child shows interest in its surroundings, examines its toys	Yes	no
Attention	Is able to focus	The child watch something or listen for more than one minute	Yes	no
	Maintain concentration	The child is able to examine toys for more than two minutes	Yes	no
	Easily distracted	The child is distracted by sounds, lights, movements, even while playing and does not return to its original activity	Yes	no
Motor activity	Generally increased level of activity	The child is characterized by a high level of activity restlessness	Yes	no
	Generally reduced level of activity	The child has a passive motoric, is mainly inactive	Yes	no
	Impulsiveness	The child is unpredictably active, throws things suddenly	Yes	no
Social communication and interaction	Eye contact	The child is able to establish eye contact. The Visiting Nurse is not in doubt that the child sees her eyes	Yes	no
	Contact smile	The child smiles to the Visiting Nurse when eye contact is made	Yes	no
	Proximity seeking	The child seeks contact with smiling, chattering, touching or reaching out after its parents	Yes	no
	Mutual communication	The child uses gestures, smiles and chatter with its parents for more than two communication loops (answer > <reply)	Yes	no
	Joint attention	The child pays attention to parents’ indications, checks and looks again	Yes	no
	Bodily contact	The child shows interest in bodily contact by expression and gesture	Yes	no
	Selectivity	The child clearly prefers the familiar care-personnel	Yes	no
Language	Language understanding	The child reacts to gestures/and some words	Yes	no
	Verbal expression	The child expresses itself with facial expressions, gestures, pointing, chatter in syllables	Yes	no

The items are answered with yes or no. A dichotomized response option was chosen to facilitate the daily use. This is in accordance with existing national health recommendations since the CHN is obliged to consider intervention when child problems of health or development are identified. To increase the CHN’s attention during the assessment, 17 of the items were formulated as fulfilling the developmental approach and 10 of the items formulated as shortcoming [24].

### Pilot study

The pilot study was conducted from January to April 2010. It included a total of 400 children who were assessed by 20 CHNs as part of the scheduled routines at home visits at child age 9–10 month. The CHNs were trained in the use of the measure by the community health head nurse and a child psychiatrist (AMS). The practical procedure was planned in dialogue with the CHNs, with the priority not to change the routines of the CHNs.

The initial face validity and feasibility of the measure was explored in three qualitative data sources: 1) semantic recordings of CHNs’ descriptions of any problem related to the screening questionnaire and the manual, based on 400 assessments, 2) feedback from CHNs in two half days joint meetings, and 3) finally, CHNs answered a questionnaire on their overall experiences regarding the face validity and feasibility of the measure. Results of the analysis of the qualitative data above resulted in minor precisions and semantic adjustments of the measure and manual, now called the Copenhagen Infant Mental Health Screening, CIMHS.

### The validity and feasibility study

The study population in this part of the study was a total of 3263 infants who were consecutively enrolled for participation in the period from 1^th^ of March 2011 to 31^th^ of December 2013. These children were recruited from the same 11 municipalities as the pilot study and included for participation as part of the home visit scheduled at child age 9–10 months. Practical procedures at the home visit were unchanged compared to former practise at home visits at child age 8–10 months, apart from the assessment according to the CIMHS at the end of the visit. As in existing routines the assessment of the child was based on the CHN’s observations and information from the parents.

Infants born before week 36 were included while adjusting for the gestational age of the child.

Excluded were infants having severe somatic and developmental disorders, and infants of parents who did not speak or understand Danish language.

Overall, a total of 45 CHNs participated, with some replacements during the study period. Prior to the study, the CHNs’ were trained in use of the measure by the community health head nurse or the principal investigator (PI). Compliance of the CHNs was optimized during the study period by supervision ad hoc and two joint seminars.

### Data

Data were obtained from the Danish Medical Birth Register (MBR), the Child Health Database (CHD) and from the CIMHS assessment at 9–10 months.

#### The Danish Medical Birth Register (MBR)

The MBR includes information on child and family factors recorded during pregnancy, birth and peri-natal period [25]. We applied the following variables from the MBR: Gestational age (coded into <32, 32- < 37 and ≥37 weeks) and a dichotomized version premature birth (<37 weeks) vs. not premature birth. Birth weight (coded into <1500, 1500- < 2500, ≥2500 g) and a dichotomized version low birth weight (<2500 g) vs. high. The variable on neonatal complications was constructed on MBR variables on any ICD-10 diagnoses of asphyxia, neonatal sepsis and/or if the child had received neonatal respirator treatment. The variable was dichotomized in any neonatal complications vs. none. Low Apgar score (<10 scores at 5 min) vs. high (Apgar score index the overall physical condition of the child in the first 10 min of birth) [26]. Mother’s smoking in pregnancy yes/no, parent’s age at child birth was dichotomized in parents young at child birth (both parents <20 years) vs. older (one or both parents ≥20 years). Parent’s place of birth was dichotomized in parents born outside Scandinavia (Denmark, Greenland, the Faroe Islands, Iceland, Norway, Sweden or Finland) vs. one or both parents born in Scandinavia. Finally, a variable of family structure was applied, parents living together at the time of the birth of the child vs. parents not living together.

#### The child health database

The database includes the CHN’s recordings from home visits during the first year of the child’s life. At each visit, the CHN record information from parents together with the results of her examinations of the child and her evaluation of the relation between parent and child. We included the following variables: Parent’s years of schooling, dichotomized in both parents ≤10 years vs. one or both parents >10 years, mother’s mental health problems recorded between child birth and child age six months, dichotomized in problems vs. no problems. Mother-child relationship recorded between child birth and child age six months, dichotomized in problems vs. no problems. Maternal mental health problems and mother-child relationship were assessed by CHNs at home visits at child age 1–4 weeks, 2–3 months and 4–6 months. These variables were recoded to be present, if the CHN had recorded problems at least at one visit. Recordings of mother’s mental health are based on mother’s information [15]. The recording of the mother-child relation is based on the CHN’s overall evaluation of information and the CHN’s observation at home visits. The evaluation thus includes standardized recordings regarding the mother’s expectations to the child, and the CHNs’ observation of the mother in interaction with the child [15].

#### The CIMHS assessment

The 27 items are shown in Table 1 The items were coded with a one for problem scores and zero for no problem.

### Analyses

Qualitative data from the pilot study were used to examine the face validity of the overall conceptualisation of mental health problems in the CIMHS. All comments in the data sources were analysed by the first stages of the Grounded Theory analyses. These analyses include an open coding, a categorisation of the comments and an interpretative reading [27].

Statistical analyses were carried out using the SAS version 9.3. Descriptive statistics was used to examine the differences between participants and non-participants regarding child sex, gestational age, birth weight, Apgar score, parent young at child birth, parent born outside Scandinavia, parent more than 10 years of schooling, parent living together at child birth, mother mental health problems and mother-child relationship. Moreover, we examined the frequency of CIMHS problems and differences between boys vs. girls, children born preterm (<37 weeks) vs. term, low birth weight (<2500 g) vs. high, low Apgar score vs. high (statistical testing by chi^2^ test, *p*-value <0.05). Exploratory factor analysis (EFA) was applied for a tentative search of patterns initially supporting the theoretical construct of infant psychopathology [24].

## Results

A total of 3263 children were eligible for the study, of which civil registration number was missing for ten, leaving 3253 children to be included in the study. A total of 280 children did not participate or were excluded, because the parents declined (*n* = 48), the child had physical or developmental illness or handicap (*n* = 15), parents did not speak or understand Danish language (*n* = 34), practical reasons (*n* = 105), not eligible (*n* = 39) and other reasons (*n* = 39). More non-participants had parents born outside Scandinavia; parents with less than 10 years of schooling and problems of mother-child relationship recorded by CHNs between child age 0–6 months, whereas no significant differences were seen with regard to child gender and perinatal adversities (Table 2).

**Table 2:** Characteristics of the study population (*N* = 3253)

	Participants	Non-participants	
	% (n)	% (n)	*p*-value (missing)
Sex			
Boys	51.7 (1538)	51.4 (144)	
Girls	48.3 (1435)	48.6 (136)	.93 (0)
Gestational age			
< 28 weeks	0.1 (2)	0.0 (0)	
28-31 weeks	0.7 (19)	0.4 (1)	
32-36 weeks	5.1 (144)	4.5 (12)	
> = 37 weeks	94.2 (2680)	95.1 (254)	.88 (141)
Birth weight			
< 1500 g	0.4 (11)	0.4 (1)	
1500 - <2500 g	4.2 (119)	2.7 (7)	
> = 2500 g	95.5 (2738)	97.0 (256)	.50 (121)
Neonatal complications			
Yes	21.3 (632)	22.5 (63)	
No	78.7 (2341)	77.5 (217)	.63 (0)
Apgar score			
< 10	5.8 (166)	4.5 (12)	
> = 10	94.2 (2697)	95.5 (254)	.39 (124)
Parental factors			
Mother smoking in pregnancy			
Yes	12.2 (350)	14.9 (39)	
No	87.8 (2510)	85.1 (222)	.21 (132)
Parent young at child birth			
< 20 years	0.4 (11)	0.0 (0)	
> = 20 years	99.6 (2886)	100.0 (270)	.31 (86)
Parent born outside Scandinavia			
Yes	27.2 (808)	48.9 (137)	
No	72.8 (2165)	51.1 (143)	.0001 (0)
Parent more than 10 years of schooling			
No	9.5 (175)	18.3 (28)	
Yes	90.5 (1672)	81.7 (125)	.0005 (1253)
Parents living together at child birth			
No	7.3 (206)	8.4 (21)	.55 (192)
Yes	92.7 (2604)	91.6 (230)	
Mother mental health problems			
Yes	27.1 (685)	23.9 (53)	
No	72.9 (1843)	76.1 (169)	.30 (503)
Mother-child relationship problems			
Yes	8.7 (221)	13.5 (30)	
No	91.3 (2313)	86.6 (193)	.02 (496)

Table 3 shows the frequency of infant mental health problems stratified on gender. Overall, problems of eating were identified in 20.1% of the children and problems of emotional regulation in 14.3%, attention problems in 15.9% and problems of communication and interaction in 20.7% of the children. A total of 17.5% of the children had three or more problems.

**Table 3:** The frequency of mental health problems in 9–10 month old infants identified by the CIMHS (*N* = 2973)

		Boys	Girls	*p*-value
		% (n)	% (n)	
Sleep regulation	Stable sleeping pattern	5.2 (80)	5.6 (81)	0.620
	Falling asleep time	2.4 (36)	2.8 (40)	0.443
	Interrupted sleep	4.5 (68)	3.9 (57)	0.525
	*Any sleeping problem (≥1 problem)*	*9.9 (152)*	*10.2 (147)*	*0.744*
Eating	Appetite regulation	5.8 (88)	7.2 (102)	0.122
	Eats too little	8.6 (131)	11.7 (168)	0.005*
	Refusal to eat	8.1 (123)	11.4 (163)	0.002*
	Vomiting without otherwise being ill	3.2 (49)	2.8 (41)	0.609
	*Any eating problem (≥1 problem)*	*18.6 (286)*	*21.6 (310)*	0.041*
Emotional regulation	Generally happy and satisfied	1.0 (16)	1.2 (17)	0.709
	Often irritable, fussy, dissatisfied	14.7 (224)	12.2 (174)	0.045*
	Cries often	1.8 (27)	1.5 (22)	0.638
	Emotionally blunted	0.4 (6)	0.5 (7)	0.692
	*Any emotional problem (≥1 problem)*	*15.4 (237)*	*13.1 (188)*	*0.074*
Curiosity and interest Attention	Curiosity, exploring	0.1 (1)	0.1 (1)	0.963
	Is able to focus	1.2 (19)	1.0 (14)	0.504
	Maintain concentration	3.3 (51)	1.6 (23)	0.003*
	Easily distracted	16.6 (244)	13.8 (192)	0.038*
	*Any attention problem (≥1 problem)*	*17.7 (272)*	*14.9 (214)*	0.041*
Motor activity	Generally increased level of activity	6.2 (94)	5.0 (72)	0.185
	Generally reduced level of activity	1.0 (15)	0.9 (13)	0.848
	Impulsiveness	4.1 (63)	4.3 (61)	0.825
	*Any motor activity problem (≥1 problem)*	*10.2 (157)*	*9.4 (135)*	*0.464*
Communication and Interaction	Eye contact	0.1 (1)	0.1 (2)	0.522
	Contact smile	1.1 (17)	1.2 (17)	0.834
	Proximity seeking (parents)	0.4 (6)	0.3 (4)	0.598
	Mutual communication (parents)	3.5 (52)	2.2 (30)	0.035*
	Joint attention	16.9 (253)	15.8 (218)	0.433
	Bodily contact	0.9 (13)	0.6 (9)	0.485
	Selectivity	4.5 (68)	2.3 (32)	0.001*
	*Any interaction problem (≥1 problem)*	*22.2 (341)*	*19.2 (275)*	0.043*
Language	Language understanding	5.0 (76)	4.6 (66)	0.627
	Verbal expression	6.2 (94)	4.9 (69)	0.121
	*Any language problem (≥1 problem)*	*10.1 (156)*	*8.3 (119)*	*0.082*
Percent with three or more problems		17.7 (272)	17.3 (248)	0.773

*CIMHS* Copenhagen Infant Mental Health Screening. * *p*-value <0.05

Significant gender differences were seen. Problems of communication and interaction and problems of attention were more common among boys and eating problems more frequent among girls (Table 3).

Analyses of differences among children born preterm vs. term, low birth weight vs. high and low Apgar score vs. high (Table 4) showed that preterm born children had overall more CIMHS problems, with significant higher prevalence of the following variables: Interrupted sleep, often irritable, fuzzy, dissatisfied, generally reduced level of activity and problems of language understanding. Differences among children with low birth weight vs. high birth weight were seen for the variables: Vomiting without otherwise being ill and language understanding. No differences in prevalence of CIMHS problems were seen for children with low Apgar score vs. high (Table 4).

**Table 4:** The frequency of mental health problems (CIMHS) in children born preterm (<37 weeks), low birth weight (<2500 g) and low Apgar score (<10 scores at 5 min) reference group children without mental health problems (*N* = 2973)

		Preterm	*p*-value	Low birth weight	*p*-value	Low Apgar score	*p*-value
		% (n)		% (n)		% (n)	
Sleep regulation	Stable sleeping pattern	7.4 (12)	0.23	3.7 (5)	0.40	5.5 (9)	0.93
	Falling asleep time	1.8 (3)	0.60	1.5 (2)	0.43	0.6 (1)	0.11
	Interrupted sleep	1.2 (2)	0.05*	2.2 (3)	0.25	1.8 (3)	0.12
	*Any sleeping problem (≥1 problem)*	*7.9 (14)*	*0.58*	*4.7 (7)*	*0.06*	*6.6 (11)*	*0.15*
Eating	Appetite regulation	5.5 (9)	0.57	8.2 (11)	0.44	5.5 (9)	0.54
	Eats too little	6.1 (10)	0.07	9.6 (13)	0.84	6.1 (10)	0.07
	Refusal to eat	5.5 (9)	0.06	6.0 (8)	0.13	19.9 (18)	0.59
	Vomiting without otherwise being ill	5.5 (9)	0.07	6.0 (8)	0.05*	2.4 (4)	0.60
	*Any eating problem (≥1 problem)*	*16.3 (29)*	*0.45*	*20.7 (28)*	*0.87*	*18.7 (31)*	*0.62*
Emotional regulation	Generally happy and satisfied	2.4 (4)	0.08	0.7 (1)	0.72	1.2 (2)	0.80
	Often irritable, fussy, dissatisfied	18.9 (31)	0.04*	14.9 (20)	0.64	14.5 (24)	0.73
	Cries often	1.2 (2)	0.67	1.5 (2)	0.91	1.2 (2)	0.67
	Emotionally blunted	0.0 (0)	0.41	0.0 (0)	0.46	0.6 (1)	0.64
	*Any emotional problem (≥1 problem)*	*18.5 (33)*	0.03*	*15.6 (21)*	*0.70*	*15.1 (25)*	*0.81*
Curiosity and interest Attention	Curiosity, exploring	0.0 (0)	0.80	0.0 (0)	0.75	0.0 (0)	0.73
	Is able to focus	1.2 (2)	0.95	1.5 (2)	0.71	1.8 (3)	0.42
	Maintain concentration	3.1 (5)	0.66	3.0 (4)	0.74	3.0 (5)	0.67
	Easily distracted	16.8 (27)	0.56	12.2 (16)	0.34	13.8 (22)	0.61
	*Any attention problem (≥1 problem)*	*15.7 (28)*	*0.73*	*11.9 (16)*	*0.16*	*14.5 (24)*	*0.51*
Motor activity	Generally increased level of activity	4.2 (7)	0.40	4.5 (6)	0.54	4.3 (7)	0.42
	Generally reduced level of activity	3.0 (5)	0.003*	1.5 (2)	0.51	1.8 (3)	0.24
	Impulsiveness	1.9 (3)	0.15	3.0 (4)	0.53	4.3 (7)	0.90
	*Any motor activity problem (≥1 problem)*	*7.9 (14)*	*0.61*	*8.9 (12)*	*0.73*	*10.2 (17)*	*0.84*
Communication and Interaction	Eye contact	0.0 (0)	0.73	0.0 (0)	0.70	0.0 (0)	0.67
	Contact smile	1.2 (2)	0.95	0.8 (1)	0.65	1.8 (3)	0.42
	Proximity seeking (parents)	0.0 (0)	0.46	0.0 (0)	0.48	1.2 (2)	0.46
	Mutual communication (parents)	2.6 (4)	0.89	3.1 (4)	0.83	3.1 (5)	0.80
	Joint attention	19.2 (30)	0.37	16.9 (22)	0.90	14.3 (23)	0.43
	Bodily contact	1.2 (2)	0.51	1.5 (2)	0.33	1.2 (2)	0.51
	Selectivity	3.8 (6)	0.83	3.8 (5)	0.86	3.7 (6)	0.86
	*Any interaction problem (≥1 problem)*	*21.4 (38)*	*0.43*	*21.5 (29)*	*0.86*	*18.1 (30)*	*0.36*
Language	Language understanding	13.0 (21)	<0.0001*	11.3 (15)	0.0004*	3.7 (6)	0.48
	Verbal expression	7.9 (13)	0.19	8.2 (11)	0.18	7.3 (12)	0.31
	*Any language problem (≥1 problem)*	*16.3 (29)*	0.0001*	*14.8 (20)*	0.02*	*10.8 (18)*	*0.48*
Percent with three or more problems		23.3 (38)	0.07	20.9 (27)	0.46	16.9 (28)	0.79

*CIMHS* Copenhagen Infant Mental Health Screening. * *p*-value <0.05

The results of the exploratory factor analyses (EFA) are shown in Table 5. EFA identified 11 items that cluster into five factors (all factor loadings >0.37): Factor 1) eating problems, factor 2) emotional problems, factor 3) attention problems, factor 4) problems of language and communication and factor 5) problems of proximity seeking and body contact (Table 5). None of these items have loading in more than one cluster. The remaining items did not fit the factor structure as the factor loadings were low.

**Table 5:** The factor structure of the CIMHS illustrated by factor loadings, *N* = 2973

	Factor1	Factor2	Factor3	Factor4	Factor5
Eat too little	**.58**	.06	.04	.01	-.03
Refusal to eat	**.57**	.08	.06	.02	.05
Generally happy and satisfied	.03	**.43**	.01	.09	.06
Often irritable, fussy, dissatisfied	.10	**.39**	.20	.06	.03
Cries often	.11	**.46**	.06	-.01	.00
Is able to focus	.02	-.05	**.37**	-.01	.04
Maintain concentration	.03	.01	**.40**	.05	.06
Proximity seeking (parents)	.03	-.03	.00	.07	**.45**
Mutual communication (parents)	.05	.03	.05	**.40**	.14
Bodily contact	.04	.01	.10	.04	**.44**
Verbal expression	.03	-.01	.07	**.44**	.15

*CIMHS* Copenhagen Infant Mental Health Screening. **Bold**: Factor loadings >0.37

### Face validity and feasibility

Qualitative evaluation among CHNs showed that the screening measure was feasible within the existing routines at CNHs’ home visits at child age 9–10 months, and considered by CHNs to be highly relevant in the communication with parents about the development and mental health functioning of the child.

## Discussion

The new screening measure CIMHS builds on solid evidence on early developmental psychopathology and the potentials of mental health screening in early childhood [2, 8]. The earliest possible valid identification of mental health problems, which is feasible within existing service settings, was a priori suggested to be at child age of 9–10 months [8]. The CIMHS was designed to be short and feasible in existing routines of CHNs, while comprehensive regarding the spectrum of mental health problems seen in children below the age of 12 months.

In a general population sample of 2973 infants, the measure identified problems of feeding and eating, sleep, developmental and socio-emotional problems in 9–10 months olds with a frequency in line with findings from other general population samples at comparable ages [8, 28, 29]. A total of 17.5% of the infants had three or more problems; and significant differences between boys and girls were seen regarding neuro-developmental problems being more common in boys, and feeding and eating problems being more common in girls. These findings are in line with prevalence studies of older preschool children [28–31]. Moreover, the findings on differences in prevalence of CIMHS items of sleep regulation, feeding and eating, reduced level of activity, and impressive language development in infants born preterm, are in line with existing evidence on a higher risk of immature regulation and developmental delays in children born premature [32, 33]. Similarly, CIMHS identified higher frequencies of eating problems manifested as vomiting without being ill, as well as problems of impressive language in infants born with low birth weight [33, 34]. These observations suggest that the CIMHS has acceptable discriminatory validity [24].

Exploratory factor analyses suggest five clusters of mental health items: 1) problems of language and communication, 2) problems of attention, 3) emotional problems, 4) problems of attachment and 5) problems of eating. The clusters identified correspond to patterns of clinical problems seen in referred children, and mental health problems identified in general population studies of children aged 0–3 years [8, 24]. Some items did not fit the factor structure e.g. sleep and motor activity, which suggest that the remaining items cover other domains, or that not all items are equally important at this particular age.

To our knowledge, this is the first screening measure that covers the full range of mental health, and which is designed specifically for use within the existing service settings and targeted infants from a general population of children below 12 months. Among measures validated in non-clinical populations the Alarm Distress Baby Scale (ADBB) [35], the Brief Infant-Toddler Social and Emotional assessment (BITSEA) [20] and the Brigance Infant and Toddler Screen (Brigance) [36] do not cover the full range of mental health problems seen in children below the age of 12 months. The Ages and Stages Questionnaire (ASQ) [18] and the Parent Evaluation of Developmental Status (PEDS) [37] cover a broader range of developmental and behavioural problems. However, these measures are based on parent’s evaluation only; and they are considerable longer than CIMHS, and thus less feasible in existing primary health care settings.

The following are considered to be major strengths of the CIMHS: 1) the measure builds on current knowledge on the developmental presentation of mental health problems, 2) an important stage of child development is targeted, 3) the whole spectrum of putative psychopathology is included, and 4) information from parents as well as assessments by health professionals are included. Moreover, 5) it is a considerable strength that the measure is developed within an existing service setting and 6) validated in a large general population sample within the same setting. 7) The validity of CIMHS has been estimated by descriptive analyses using data from Danish registers regarding perinatal risk factors, as well as exploratory factor analyses. 8) Feasibility was documented by qualitative analyses of acceptability of parents and health professionals, and 8) finally, the high participation rate in the use of CIMHS suggest that the measure was well accepted by the parents.

Some limitations need to be highlighted. First, there are obvious challenges of developing a new measure, which aims to cover the full range of infant mental health problems. We are fully aware of the pitfalls of this, and we have sought to integrate all main aspects of current conceptualizations of infant mental health, and have included as many items as feasible from existing validated measures. Overall, we have taken into account, the importance of developmental variations and shifts in behaviour, skills and regulation in this young age [6]. Accordingly, the manual of the CIMHS repeatedly states, that the definition of particular deviations from a putative normative developmental course, only become problems when they occur either in excess or too infrequently [6].

It is a limitation of the study that we cannot report on concurrent validity against a gold standard, e.g. regarding overt developmental disorders. An in-depth assessment of child development might have further qualified results, however at this young age, 9–10 months, even highly standardized and validated measures, as the Bayley’s Scales of Infant Development, have questionable validity [17]. Moreover, the financial resources to perform in depth psychological and clinical assessments were neither available, nor within the scope of the present study. However, the next stages of the validation of the measures, include in-depth assessments of development and psychopathology at age 18 months; as well as planned register-follow-up regarding disorders of mental health and development diagnosed at hospitals.

Another limitation concerns the unknown feasibility and validity of CIMHS in children with severe physical illness or major developmental handicap, or children of parents who did not understand and speak Danish language, as these were not included in the present study.

Taken together, the demonstrated content validity, discriminatory validity and feasibility of the newly developed measure, CIMHS, suggest promising potentials regarding infant mental health screening in existing service settings. The validity has to be further explored regarding construct validity, reliability, sensitivity, specificity and positive and negative predictive value.

## Conclusion

The infant mental health screening measure CIMHS shows promising face and content validity. It is well accepted by parents and community health nurses, and feasible in existing general child health surveillance in the municipalities in Denmark.

Before implementation in the general child health surveillance, CIMHS has to be further psychometrically evaluated in the existing service settings. Moreover, it has to be combined with intervention that target children identified by CIMHS to have potential mental health problems.

## Abbreviations

CHD: The Child Health Database
CHN: Community health nurse
CIMHS: Copenhagen Infant Mental Health Screening
EFA: Exploratory factor analyses
MBR: The Danish Medical Birth Register.
